# Pilot study of implementing Managing and Adapting Practice in a German psychotherapy master’s program

**DOI:** 10.1038/s41598-024-67407-w

**Published:** 2024-07-16

**Authors:** Katharina Szota, Anna S. van der Meer, Teri Bourdeau, Bruce F. Chorpita, Mira-Lynn Chavanon, Hanna Christiansen

**Affiliations:** 1 Department of Psychology, Philipps-University of Marburg, Gutenbergstr. 18, 35032 Marburg, Germany; 2 Department of Psychology, Goethe-University Frankfurt, Varrentrappstr. 40-42, 60486 Frankfurt am Main, Germany; 3 PracticeWise, PO Box 372657, Satellite Beach, FL 32937 USA; 4 UCLA Department of Psychology, 1285 Franz Hall, Los Angeles, CA 90095 USA

**Keywords:** Implementation, Evidence-based practice, Mental health care, Children and adolescents, Psychotherapy training, Paediatric research, Translational research, Health services

## Abstract

Despite a significant accumulation of research, there has been little systemic implementation of evidence-based practices (EBP) in youth mental health care. The fragmentation of the evidence base complicates implementation efforts. In light of this challenge, we sought to pilot a system that consolidates and coordinates the entire evidence base in a single direct service model (i.e., Managing and Adapting Practice; MAP) in the context of a legal reform of psychotherapy training in Germany. This pilot study aimed to evaluate the feasibility of the implementation of MAP into the curriculum of the reformed German master's program. Eligible participants were students in the master’s program at Philipps-University Marburg during the winter-term 2022/2023. Students first learned about MAP through introductions and role plays (seminar 1), followed by actively planning and conducting interventions using MAP resources for patients in a case seminar under supervision (seminar 2). A repeated-measures survey was conducted to investigate students’ knowledge gains, perception of MAP and changes in their self-rated confidence to use EBP. Results indicated that students perceive MAP to be manageable to learn. Positive progress was achieved with regard to their knowledge and self-reported confidence to use EBP, although interpretation and generalization of the results are limited by small and homogeneous samples, lack of statistical power and missing comparison groups. The feasibility of the implementation and suitability of measures are discussed. Important implications could be drawn with regard to future investigations.

## Introduction

With a growing number of global crises, such as the COVID-19 pandemic, climate change, war, hunger and accompanying refugee movements, the mental health burden on young individuals and the need for professional mental health care for children and adolescents worldwide is rising^1,2^. To meet this growing demand, pragmatic and effective interventions need to be implemented within the health care system while remaining easily accessible for both patients and providers. In the last decades, psychotherapy research demonstrated that evidence-based practices (EBP) show advantages like higher effect sizes compared with usual care^3,4^ as well as increased cost-effectiveness^5–7^. EBP are defined as “the integration of best research evidence with clinical expertise and patient values”^8,9^. They are required to monitor the effectiveness or potential harms of interventions during treatment and to be “consistently science-informed, organised around client intentions, [and] culturally sensitive”^10^.

Despite a significant accumulation of research^11^, EBP are not widely represented in mental health care^12,13^, and there has been little systemic implementation of EBP for youth in mental health care systems. Implementation efforts are complicated by the rapid growth of specific evidence-based interventions and the accompanying fragmentation of the evidence base, i.e. the large number of study results that only apply to single interventions. The wealth of options that are essentially “non-interoperable” typically requires practitioners to learn a large number of independent interventions in order to manage a typical caseload^14^. Accordingly, extracting core practices and process models (e.g., overlapping practice elements) of various evidence-based interventions^15,16^ enables one to scale up EBP by enabling practitioners to engage in a harmonized, transdiagnostic, responsive approach to evidence-based intervention, with a manageable amount of workforce development activity that can be paced over time^17,18^. Moreover, this individualized approach addresses the frequently discussed challenge that EBP are not suitable to a range of patients, especially in case of comorbidity, resulting in low response rates^11,19^. Individualized approaches are characterized by tailoring and adapting psychotherapy to specific patient characteristics and situations in addition to their disorders in order to enhance treatment effectiveness^20^. Indeed, they have been shown to outperform usual care^21^ as well as “gold standard” evidence-based treatment^3,22^.

Managing and Adapting Practice (MAP) is a system that consolidates and coordinates the entire youth mental health evidence base within its direct service model. In distinction to individual EBP, MAP provides a framework, concepts and diverse resources to help providers to identify and select, personalize, implement and evaluate modular transdiagnostic interventions based on the research evidence^23^. MAP resources include the *PracticeWise Evidence-Based Services Database* (PWEBS), the Practitioner Guides summarizing either common procedures among evidence-based practices (*Practice Guides*) or frameworks for organizing service delivery (*Process Guides*), and *Clinical Dashboards*, Microsoft Excel™ based tools to visualize the treatment plan, process and progress^23^. The PWEBS database enables providers to search within a database of coded randomized controlled trials (RCTs) of youth interventions. Updates to the database are derived from two main sources: ongoing literature searches conducted by PracticeWise, and RCTs nominated by researchers, and community partners^24^. Users can customize their PWEBS search to retrieve evidence-based interventions that align with the individual youth’s problem area and characteristics. To provide a comprehensive overview of the results, the treatment families, practice elements, setting and format of the interventions are displayed with their frequencies in the available studies. Previous studies in Minnesota^25^ and Los Angeles^26,27^ demonstrate the instructional efficacy of the MAP system as well as its effectiveness in achieving large-scale and rapid implementation with considerable evidence of sustainability^28^. This might be due to the higher therapist satisfaction with and acceptance of MAP^29,30^.

Moreover, evaluations on youth outcomes of MAP implementation are promising. Southam-Gerow et al.^26^ report effect sizes ranging from *d* = 0.59 to *d* = 0.80 on a caregiver report measure of emotional and behavioral problems before and after MAP interventions. The modular approach to therapy for children with anxiety, depression, trauma, or conduct problems (MATCH-ADTC)^31^ is a specific intervention protocol designed using practice coding of four EBP as well as the MAP architecture and concepts. The intervention is comprised of 33 treatment components that are frequently included in well-supported EBP to address youth anxiety, depression, trauma and conduct problems. Comprehensive flowcharts are provided to guide the selection and arrangement of therapy procedures and step-by-step instructions facilitate the implementation of treatment components. Concurrently, service providers are supported to individualize interventions and address comorbidity and treatment interferences^31^. When compared with a county-supported implementation of multiple evidence-based practices for youth, MATCH-ADTC resulted in faster and greater improvement for children than receiving standard EBP. Moreover, the children in the MATCH-ADTC treatment condition were less likely to receive additional psychosocial treatment services or psychotropic medications^22^. Recently, an adapted MATCH-ADTC intervention for children with epilepsy was evaluated in an RCT in the UK and was found superior to assessment-enhanced usual care regarding the reduction of emotional and behavioural difficulties^32^.

In Germany, MAP is familiar only among youth mental health researchers and essentially unknown within routine practice providers or service organizations. Recently, the psychotherapy training for state-licensed professional psychotherapists began to undertake major changes aiming to foster the scientist-practitioner approach. Based on a federal legal reform, more competency-based courses and practice components are included in the university curricula to obtain the master’s degree in clinical psychology and psychotherapy. With the current educational changes comes an opportunity to contribute to the dissemination and implementation of EBP in the German mental health system. Therefore, we aim to implement MAP into the German healthcare system by incorporating it into two modules of the reconceptualized master’s degree program of clinical psychology and psychotherapy, with a total of 3 courses and 8.5 credits. Our pilot study explored the feasibility of implementing MAP in everyday university settings in Germany. We first focused on our students’ reception and perception of MAP, as feedback from future providers’ (i.e., the students) is indispensable to achieve sustainable implementation^33^. The development of students’ (self-)confidence in using EBP is a key concern across the different courses. Besides, we also aimed to monitor the quality of our teaching through effects on students' knowledge. The pilot study should inform us whether the evaluation design and its instrumentation by means of the selected questionnaires is suitable to monitor the teaching concept and the implementation of MAP. Accordingly, we aimed to draw practical implications from our pilot study for the future implementation of MAP in the German education and health care system and its empirical investigation.

## Methods

### MAP implementation

The two seminars of interest focus on training students in evidence-based psychotherapy intervention practices for children and adolescents. They consist of a first seminar with theory-based instruction of the evidence based, modular treatment approach MAP with role play practices on the interventions learned; and a second seminar enabling the application of the approach in a case class, where the students treat a group of patients under continuous live supervision by a licensed psychotherapist.

**(1) Seminar 1** started with an introduction to MAP in general with the opportunity for students to ask questions or discuss concerns. After rehearsal of MAP concepts and resources, the students practiced several interventions from the MAP Practitioner Guides portfolio in role plays (small groups of 3 to 4 students) which were supervised by a MAP instructor. Those interventions were picked as they address central foci of child and adolescent psychotherapy both from the perspective of the child/youth and the parents. Also, the introduced practice guides represent basic interventions in child and adolescent psychotherapy, which is why they are especially helpful for early professionals. Further, demonstrating how to self-learn and then to use those interventions is essential for future practice, as psychotherapists are required to continuously update their knowledge and practices. All concepts, resources and applications covered in seminar 1 are listed in Table 1.

**Table 1:** Description of MAP curriculum components adapted from Becker et al., 2022.

Component	Description
Concepts	
Evidence-based services system model	Model to guide service planning and delivery with consideration and coordination of multiple sources of evidence
The CARE Process^a^	Problem solving process of evaluating central questions, considering individual evidence, implementing identified solutions and evaluating the responses
Connect-Cultivate-Consolidate	Coordination of interventions across treatment phases
Focus-Interference Framework	Differentiation of prioritized treatment targets and anticipated interferences
Treatment planner	Creating a treatment plan by considering Connect-Cultivate-Consolidate and Focus-Interference
Session planner	Coordination of pre- and post-session activities
Resources	
PWEBS Database	Synthesis of research results on effective interventions, searchable with user-defined parameters (e.g., youth characteristics)
Clinical Dashboards	Progress monitoring system with case information, planned and implemented interventions and session feedback
Practice Guides	Summaries to guide the performance of common clinical interventions
Therapist portfolio	System to monitor the MAP learning progress
Applications	
Assessment	Generating case-specific evidence by considering multiple measures across multiple domains
Monitoring	Monitoring session-wise outcomes and feedback to compare the expected and actual progress
Planning	Planning interventions based on the case-specific information and evidence base
Practice delivery	Rehearsal and actual delivery of interventions
MAP system	Systematic and collaboration of MAP components

*Notes.* MAP,  Managing and Adapting Practice; PWEBS , PracticeWise Evidence-based Services Database; CARE,  Consider, answer, respond, evaluate.

^a^This component was only conducted in two courses.

** (2) Seminar 2** was completed in a high frequency day format setting within one week (five days with interventions from 9:30 am to 3:30 pm and preparation from 9:00 to 9:30 am and 3:30 to 6:00 pm each day) in the outpatient clinic for child and adolescent mental health care of Philipps-University Marburg, Germany. Students planned and conducted psychotherapy sessions under live supervision by licensed therapists for a group of patients (overall 4 classes with 13–16 students each and 3–5 children treated per class). Patients presented with various mental health disorders, for example attention deficit/hyperactivity or anxiety disorders. The interventions were planned with the help of the MAP resources (for example a *PWEBS* search) and conducted with the support of the *Practitioner Guides* as covered in seminar 1. Students created *Clinical dashboards* to evaluate the treatment progress.

### Pre-piloting

In winter-term 2021/22, we conducted a very first MAP implementation, evaluated students’ views on MAP and considered their verbal feedback to plan the current pilot study. In the following, we describe the adaptations that were made based on the pre-piloting.

#### Pre-piloting the implementation

Due to the Covid-19 pandemic, seminar 1 was conducted asynchronously as well as synchronously online over the semester in winter-term 2021/22. It was based on a flipped classroom concept. Accordingly, the students prepared central MAP concepts or resources by working independently and asynchronously through the MAP online training modules. In synchronous online appointments, the contents were rehearsed and interventions were rehearsed in role-plays.

In winter-term 2022/23, all appointments took place on-site rather than online, and students received more guidance. In addition, different training formats for seminar 1 were tested: While a large part of the students received a four-day intensive training, for one group of 15 students, seminar 1 was conducted over the semester. This was due to university requirements for the organization of teaching. Moreover, the German education team was supported and counseled by PracticeWise professionals in teaching a class of 49 students. Besides supervising the classes and checking the alignment with standardized PracticeWise teaching, they provided feedback on the clinical dashboards that students created in seminar 2.

#### Pre-piloting the evaluation

In our first evaluation in winter-term 2021/22, a total of 13 assessments were conducted to assess progress and changes during seminar 1 and 2. Besides students’ providing information on demographics and training, the following instruments were used: Perceived Characteristics of Intervention Scale (PCIS)^34^, Evidence Based Practice Attitudes Scale (EBPAS-36D)^35,36^, Adaptations to Evidence-Based Practices Scale (AES)^37^. In addition, students rated their agreement on visual analogue scales (VAS) providing global assessments (1) on their confidence to use EBP with six questions and (2) on their contentment with the implementation of specific interventions and adherence to MAP Practitioner Guides with six questions. The students expressed concern about the time and effort involved in this survey procedure. In addition, some of these instruments turned out to be inadequate or unsuitable during our pre-piloting, namely the PCIS subscales *Relative advantage*, *Compatibility*, *Trialability*, *Observability* and *Task issues*, the AES and the VAS on MAP implementation. This will be elaborated in more detail in the discussion section.

Accordingly, we applied a reduced survey procedure in winter-term 2022/23 with only two assessments to capture changes during seminar 2 (before and after the last day). In addition, based on the feedback of the PracticeWise professionals and in order to obtain more comprehensive data pertaining to the acquisition of knowledge and skills, we piloted knowledge assessments pre-post to the introduction of new topics in seminar 1. The 50 knowledge questions were provided by PracticeWise for research purposes and cover knowledge about the MAP system and concepts, and interventions to treat depression, anxiety, traumatic stress and disruptive behavior in youth.

### Ethics

The Internal Review Board of the Philipps-University Marburg approved the evaluation (approval number: 2021-73k). All methods were performed in accordance with the institutional guidelines. Except for the knowledge tests, participants received full study information and provided written informed consent before they were able to access the survey. Each participant created an individual code based on letters and numbers to enable repeated measurements with anonymously collected data. All raw data were stored securely at the Department of Clinical Child and Adolescent Psychology at Philipps-University Marburg, Germany.

### Participants

Eligible participants were first-year students enrolled in the reconceptualized master’s program in clinical psychology and psychotherapy of Philipps-University Marburg, Germany during the winter-term 2022/23. All students were required to complete the two seminars of interest as part of the module on professional qualification (in German: Berufsqualifizierende Tätigkeit, BQT-II) aligning to state license requirements formulated in the reformed psychotherapy law. No exclusion criteria were applied.

A total of 83 students were eligible participants. An anonymous sample of *N* = 80 (96.39%) completing seminar 1 participated in the knowledge assessments. We refrained to assess any personal and potentially identifying information in addition to the knowledge tests as not to pressure students.

Of the 83 students in seminar 1, *n* = 57 were randomly assigned to participate in seminar 2 in the same winter term. Please note that due to organizational constraints, only 60 places are available to students in seminar 2 at the end of a winter term. A further 30 places are only available to students at the end of the summer term. An employee of the organisation team, who was not informed about our survey and its objectives, was responsible for allocating places by lottery to students who would complete seminar 2 in the winter and summer semesters. Of those 57 students a sample of *N* = 36 (63.16%) completed the first evaluation on seminar 2 (assessment 9). Further details on these participants are presented in Table 2.

**Table 2:** Sample description.

	*N/M*	%/SD
Eligible students	57	100.00
Participating students	36	63.16
Age	23.14	1.25
Range	20–26 years	
Gender		
Female	34	94.40
Male	1	2.80
Diverse	1	2.80
Practical clinical experience (e.g., due to internships)		
None	0	0.00
Up to 3 months	16	44.40
4–6 months	16	44.40
7–9 months	2	5.60
10–12 months	1	2.78
More than 12 months	1	2.78

A total of *n* = 13 (22.81%) completed the second evaluation (assessment 10) after seminar 2. No significant differences were found between students that completed both assessments and those that only completed the first evaluation regarding their age (*U* = 144.50, *p* = 0.871), confidence in using EBP (*U* = 123.50, *p* = 0.397), PCIS subscales *Complexity* (*U* = 58.00, *p* = 0.999), *Potential for reinvention* (*U* = 20.00, *p* = 0.397), *Nature of knowledge* (*U* = 28.00, *p* = 0.059), and *Technical support* (*U* = 49.00, *p* = 0.999) and EBPAS-36D (*U* = 98.00, *p* = 0.535) at the first evaluation.

### Data collection

**Figure 1:**
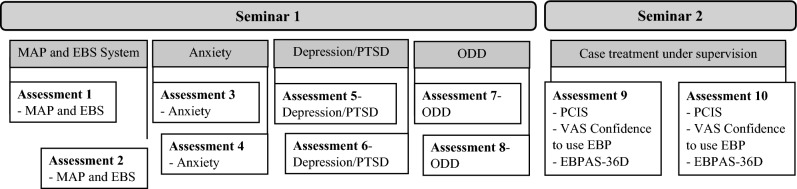
Flow charts of the assessments. *Notes.* EBP, Evidence Based Practices; EBPAS-36D,  Evidence Based Practice Attitudes Scale; PCIS, Perceived Characteristics of Intervention Scale; PTSD, Posttraumatic stress disorder; VAS, Visual Analogue Scale.

Repeated measures data were collected via an openly accessible online survey, using the scientific survey platform SoSci Survey (www.soscisurvey.de). The link was distributed via e-mail to all eligible participants. Participation was voluntary without compensation for students’ efforts. Figure 1 displays the data collection procedures.

### Measures

#### Knowledge tests

50 standardized knowledge items from PracticeWise were used to assess students’ knowledge of MAP and the EBS system and the treatment of specific mental disorders (i.e., depressive disorders, anxiety disorders, trauma disorders, and disruptive disorders) in children and adolescents. Items were questions with four to five answers of which one right answer had to be chosen. Individuals’ scores can have values ranging from 0 to 100 in increments of ten, representing the percentage of correct answers.

#### Demographics and information on training

Participants provided standard demographic and training information (e.g., age, semester) in the first survey of seminar 2.

#### Perceived Characteristics of Intervention Scale (PCIS)

The PCIS *subscales Complexity*, *Potential for reinvention*, *Nature of knowledge* and *Technical support* were used to evaluate students’ views on MAP (see Fig. 1). The PCIS was developed by Cook, Thompson and Schnurr^34^ to assess health care providers’ views of interventions. The authors found it to be a reliable measure of perceived characteristics of particular evidence-based treatments for mental health care. Respondents are asked to rate their agreement with statements on a 5-point Likert scale ranging from 1 (‘not at all’) to 5 (‘a very great extent’). All items are worded in such a way that higher scores indicate more positive evaluations of the intervention. The items load on subscales with two items each^34^: The subscale *Complexity* (internal consistency in the current sample: α = 0.37) assesses the level of difficulty to understand and use the innovation and the subscale *Potential for reinvention* (α = 0.87) the ability to refine, elaborate and modify the innovation. The subscale *Nature of knowledge* (α = 0.91) captures the amount of knowledge and skills that are required to implement the innovation and the subscale *Technical support* (α = 0.91) inquires whether the manual or material is helpful. The German translation of the PCIS can be found in Table S1 in Supplemental material 1.

#### Global assessments on confidence to use EBP

Students rated their own confidence and competence in using EBP on visual analogue scales (from 1 to 101) for six questions, e.g. ‘To what extent do you feel confident in using EBP?’ Since the total scale shows good internal consistency with Cronbach’s alpha α = 0.92-0.98 in the current sample, a composite mean score was calculated. Higher scores indicate greater confidence. All items can be found in Table S2 in Supplemental material 1.

#### Evidence Based Practice Attitudes Scale (EBPAS-36D)

The EBPAS-36D was used to assess students’ attitudes toward adopting EBP. The German translation of the EBPAS-36^34^ has been psychometrically investigated in a German-speaking sample of mental health care providers^36^. The 36 items assess positive attitudes toward EBP (e.g., their fit with values and needs of providers and patients) as well as ambivalent attitudes (e.g., the burden of learning EBP). Respondents are asked to rate their agreement with statements on a 5-point Likert scale ranging from 0 (‘not at all’) to 4 (‘to a very great extent’). Most items are worded in such a way that a higher total score indicates a more positive attitude towards the adoption of EBP; 15 items are scored reversely. A mean of the subscales can be computed to create a total scale (internal consistency in the present sample at the first assessment: α = 0.88).

### Statistical analysis

All statistical analyses were performed using IBM SPSS 28.0.1.1 for Windows (Chicago, IL, USA). To obtain internal reliability coefficients of the scales and subscales, Cronbach’s alpha was calculated. Values above 0.70 are regarded as acceptable, higher than 0.80 as good, higher than 0.90 as excellent. Nonparametric tests were used due to lack of normal distributions and small sample sizes. Mann–Whitney-U-Tests were used to assess differences between participants that answered both surveys on seminar 2 and those that dropped out. Wilcoxon-tests for paired samples were used to investigate changes from before to after seminar 1 and before to after seminar 2. *P* values < 0.05 were set as thresholds for statistical significance.

Regarding the knowledge changes, we first evaluated differences in the knowledge scores before vs. after seminar 1. In the following, we compared the three separate seminar groups: one group that received a four day intensive training with support of international PracticeWise professionals (*high frequency with counseling*, *n* = 49), one group that received one-week intensive training by our German team alone (*high frequency*, *n* = 15) and the last group that visited seminar 1 over the course of the semester, also conducted by the German team (*low frequency*, *n* = 15). As changes in students’ perceptions of MAP and their knowledge were investigated with four subscales each, we corrected the alpha level using the Bonferroni procedure (α = 0.013) for these analyses to reduce the risk of familywise error due to multiple testing.

### Ethics approval statement

The Internal Review Board of the Philipps-University Marburg approved the first evaluation (approval number: 2021-73 k).

### Participant consent statement

Except for the knowledge tests, participants received full study information and provided written informed consent before they were able to access the survey.

## Results

### Knowledge

When comparing students’ scores on the knowledge tests before and after training days of seminar 1, significant differences were found regarding students’ knowledge of MAP and the EBS system; *Z* = − 6.12; *p* < 0.001; anxiety disorders; *Z* = − 5.37; *p* < 0.001; posttraumatic stress disorders (PTSD) and depression; *Z* = − 4.44; *p* < 0.001; and oppositional defiant disorders (ODD); *Z* = − 7.35; *p* < 0.001 (see Table 3).

**Table 3:** Wilcoxon-tests for paired samples (seminar 1).

	Total sample (*N* = 79)		High frequency with counseling (*n* = 49)		High frequency (*n* = 16)		Low frequency (*n* = 15)	
Knowledge tests	*Z*	*p*	*Z*	*p*	*Z*	*p*	*Z*	*p*
MAP and EBS system^b^	− 6.12	< 0.001*	− 5.44	< 0.001*	− 3.22	< 0.001*	− 0.95	0.362
Anxiety^b^	− 5.37	< 0.001*	− 4.50	< 0.001*	− 2.49	0.018	− 1.81	0.090
PTSD and Depression^b^	− 4.44	< 0.001*	− 2.07	0.039	− 3.33	< 0.001*	− 2.50	0.013
ODD^b^	− 7.35	< 0.001*	− 5.87	< 0.001*	− 2.72	0.005*	− 3.47	< 0.001*

*Notes.* ^b^Bonferroni-adjusted α = 0.013.

*Significant result of two-sided exact test.

For the high frequency group with counseling, significant differences between the knowledge scores before and after the seminar emerged for the MAP and EBS system, anxiety disorders and ODD, but not for PTSD and depressive disorders; *Z* = − 2.07, *p* = 0.039. For the high frequency group without counseling, significant differences between the knowledge scores before and after the seminar emerged for the MAP and EBS system, PTSD and depressive disorders and ODD, but not for anxiety disorders; *Z* = − 2.49, *p* = 0.018. For the low frequency group, significant differences between the knowledge scores before and after the seminar emerged only for ODD, but not for the MAP and EBS system, PTSD and depressive disorders and anxiety disorders (see Table 3).

### Evaluation of MAP

Characteristics of MAP were rated as acceptable at the start of seminar 2 with *M* = 3.80 (*SD* = 0.63) on the PCIS subscale *Complexity*, *M* = 4.13 (*SD* = 0.79) on the PCIS subscale *Potential for reinvention*, *M* = 3.64 (*SD* = 0.76) on the PCIS subscale *Nature of knowledge* and *M* = 3.48 (*SD* = 1.09) on the PCIS subscale *Technical support*. They remained stable during seminar 2 as no significant differences emerged comparing scores before and after the seminar (see Table 4).

**Table 4:** Wilcoxon-tests for paired samples (Pre/post) for Seminar 2.

Scale	*Z*	Two-sided exact *p*
PCIS Subscales		
Complexity^b^	− 0.32	0.844
Potential for reinvention^b^	− 1.30	0.375
Nature of knowledge^b^	− 2.06	0.063
Technical support^b^	− 0.11	0.938
Visual analogue scale		
Confidence to use EBP	− 2.76	0.003*

*Notes.* PCIS, Perceived Characteristics of Intervention Scale; EBP, Evidence-based practices.

^b^Bonferroni-adjusted α = 0.013.

*Significant result.

### Confidence in using EBP

Significant higher scores were reached after seminar 2 compared to before; *Z* = − 2.76,* p* = 0.003; indicating increasing confidence in students’ EBP use.

### Attitudes towards EBP

Students reported moderate attitudes towards EBP on the EBPAS-D36, *M* = 2.92 (*SD* = 0.27). No significant changes emerged between their ratings before and after seminar 2; *Z* = 35.50, *p* = 0.824.

## Discussion

The present study aimed to pilot the implementation of MAP into the reformed master’s degree program in clinical psychology and psychotherapy in Germany and to evaluate the feasibility of our study design and methods. To discuss our results, we will first discuss our implementation procedures and results on students’ knowledge changes, evaluation of MAP, and confidence in using EBP. Thereafter, results on the feasibility of research methods are discussed and lessons learned are summarized. After reviewing major limitations of our pilot study, we aim to conclude practical implications for the future implementation of MAP in the German educational and health care system.

### Lessons learned on the MAP implementation

The first cohorts of students at Philipps-University Marburg completing the BQT-II module of the reconceptualized master’s program were instructed in the use of MAP, first through theoretical seminar content and role plays in small groups (seminar 1), and finally by planning and conducting interventions for a group of patients in a case seminar under supervision (seminar 2).

To capture the quality of our teaching, students answered knowledge tests before and after class. The results indicate an increase in expertise. Hereby, we compared three different training formats (high frequency with counseling, high frequency and low frequency). Although the results should be interpreted with caution due to the small and very different sample sizes across the training conditions, it seems that carrying out the seminar over the course of the semester was inferior to a high frequency format. Until now, few studies have evaluated the optimal approaches of therapist trainings to implement EBP. One systematic review indicates that more intensive training approaches that go beyond provision of manuals and brief workshops but provide additional components like consultation are more effective^38^. However, the optimal number, duration and spacing of training sessions requires further investigation. As Henrich, Glombiewski and Scholten^39^ point out, the distribution of sessions over a longer period of time might allow therapists to expand and consolidate the acquired skills by applying them in their clinical practice between sessions.

Students in our pilot study evaluated the MAP system as quite understandable, easy to use and manageable to learn as well as equipped with helpful materials. Thus, it can be assumed that the students felt capable of learning contents and competencies they assumed to be important for the implementation of MAP. The average ratings were comparable to those reported by Cook et al.^34^ and Stadnick et al.^40^. Encouragingly, it appeared that students’ confidence in being able to use EBP increased during seminar 2. This is of relevance as results of previous studies indicate that practitioners’ self-rated confidence, competence or perceived behavioral control to use EBP predicts their intention or actual use of interventions^41,42^. At the same time, research evidence indicates that therapists tend to overestimate their interventions’ effectiveness^43^, and have limited competencies to predict negative treatment outcomes^44,45^. Accordingly, education in the master’s program of clinical psychology and psychotherapy should aim to enhance students’ perceived capability to implement EBP while at the same time improving their ability to anticipate adverse treatment processes in order to make adjustments. Continuous progress and outcome monitoring during treatment and the collaborative evaluation of the data with supervisors is not yet common practice, but would likely improve patient outcomes^46,47^. As a result of such positive processes, practitioners’ perceived confidence can presumably be enhanced as well.

To summarize, the implementation of MAP into the reformed master's degree program in clinical psychology and psychotherapy is feasible. Students’ valuable feedback will hopefully help us to enhance their skill building and implementation sustainment. In the future, students’ abilities to monitor treatment processes might be encouraged by incorporating client and supervisor feedback.

### Lessons learned on the implementations’ evaluation

Another goal of our pilot study was to evaluate the feasibility of the study design and the suitability of measures in the target group. Major difficulties emerged with regard to the measures already during our pre-piloting. The majority of the available instruments usually used in dissemination research seemed unsuitable for our context, which did not involve the implementation of specific, manualized EBP. These are important observations, given that one goal of our pilot implementation study was to determine which measures might be appropriate for a larger implementation evaluation (see^48^).

Firstly, the PCIS subscales *Relative advantage*, *Compatibility*, *Trialability*, *Observability* and *Task issues* revealed to be inadequate as they expect a comparison with the usual practical activity or other treatments. Understandably, our students gave us the feedback that they do not have enough practical experience that would allow these comparisons. Internal consistencies of the subscales indicate inadequate reliability, perhaps due to the limited ability of students to provide answers. The remaining four subscales *Complexity*, *Potential for reinvention*, *Nature of knowledge* and *Technical support* seemed applicable. Moreover, we consider these scales to be relevant, as they map some of the benefits or potential drawbacks that users might consider when deciding whether they apply MAP to their therapeutic service. However, it should be noted that the subscale *Complexity* showed low internal consistency in the current sample and should be interpreted with caution.

Secondly, the EBPAS-36D that we used to assess students’ attitudes towards EBP must be critically reflected. Although the EBPAS-36 is a highly relevant instrument for implementation science with good psychometric properties^35^, its definition of EBP and associated advantages and disadvantages fits better with specific and often manualized interventions than with the flexible and modularized application of MAP as a comprehensive system. Some of the items therefore seem inappropriate to capture attitudes towards the use of MAP or modularized psychotherapy, for example “Clinical experience is more important than using manualized therapy/treatment”. Modifications may allow capturing attitudes toward modularized psychotherapy and MAP^49,50^. This causes ambiguity since reservations about manualized EBP may indicate a preference for the modularized approach in MAP. The Modified Practice Attitude Scale (MPAS,^49^) might represent an alternative to the EBPAS being a revised version without referencing “manualized” interventions.

Thirdly, we used the AES in our pre-piloting to assess adaptations that were made by the student groups when implementing MAP during practical training in seminar 1 and seminar 2. The AES might be used to capture providers’ “adherence” to a manualized treatment protocol, with fewer adaptations ascribed to a more adherent, more desirable behavior. Usually, adjustments to the practical approach would be considered lack of adherence. However, our goal is to enable students to plan and implement interventions that are as individualized as necessary. For our purpose, aiming to evaluate the implementation of MAP, quite the opposite could be argued: Few adaptations might be interpreted as having less integrity with the highly flexible and extensive approach that MAP represents. The same aspect holds true for the scale on MAP implementation that we initially used, as two items each asked students to self-rate their adherence to the Practitioner Guides.

Lastly, we used specific knowledge tests to assess changes of students’ information about MAP and the EBS system model, and the treatment of relevant mental disorders of children and adolescents. We found that our students already had a fairly high level of knowledge about the treatment of anxiety disorders before the seminar. Thus, the identification of changes could be limited by instrumental ceiling effects.

In summary, we conclude that specific assessments of relevant outcomes with pragmatic instruments should be pursued. The students’ limited prior experience should be taken into account. It would also be desirable to include external assessments, for example by the supervisor or independent evaluators. Incentives for study participation and especially completion need to be considered in view of the high drop-out rate.

### Limitations

Interpretation and generalization of the results are limited by a small and homogeneous sample of predominantly female students and quite similar levels of professional experience. Due to the small sample size there is low statistical power, so relevant changes may not have reached statistical significance or could not be assessed. This is particularly evident in light of the low survey completion rate. It cannot be ruled out that the results are biased by the self-selection of those who continued to take part in the surveys. In light of this, future evaluations should set low demands while at the same time communicate the rationale for the assessments comprehensibly. Separate analyses of the self-rated competency progress for students with more or less professional experience or moderation analyses due to different attitudes towards EBP could be performed on larger samples in the future. Last but not least, it should be taken into account that since there is no comparison sample, the changes could have been caused by other course components, personal development, or external influences. Some of the measures we used were translated by our team and should be psychometrically investigated in German samples. In addition to internal consistency, factorial validity and measurement invariance should also be investigated in the given target group.

## Conclusions

Our pilot study shows that MAP can be integrated into seminars of the BQT-II module and that knowledge and confidence gains among students and future psychotherapists can be achieved. The results help to improve further implementation of MAP into the master’s program and the German mental health care system. In upcoming semesters, the implementation of MAP will be continued and evaluated via BQT-II and BQT-III. In addition, a multicenter study is planned to investigate the effects of the implementation at different universities on the competence development of future psychotherapists and the treatment outcomes in child and adolescent outpatient clinics. Besides including assessments of patients as well as their caregivers to provide continuous feedback to our students, we plan to incorporate external assessments on the integrity of students’ implementation of MAP by evaluating their treatment plans and progress evaluations. In addition, we intend to distill relevant competencies for individualized psychotherapy and create and psychometrically investigate instruments for this purpose.

### Supplementary Information

Supplementary Tables.

## Data Availability

The datasets without potentially identifying socio-demographic and occupational information analyzed in the current study are available from the corresponding author on reasonable request.

## Abbreviations

AES: Adaptations to Evidence-Based Practices Scale
BQT: Module on professional qualification (German: Berufsqualifizierende Tätigkeit)
EBPAS-36D: Evidence Based Practice Attitudes Scale (German version)
MAP: Managing and Adapting Practice
MATCH: Modular Approach to Therapy for Children
ODD: Oppositional defiant disorder(s)
PCIS: Perceived Characteristics of Intervention Scale
PTSD: Posttraumatic stress disorder(s)
PWEBS: PracticeWise Evidence-Based Services database
RCTs: Randomized controlled trials
