# Supplementary material for: Pilot study of implementing Managing and Adapting Practice in a German psychotherapy master’s program
Source: Sci Rep. 2024 Jul 16;14:16466. doi: 10.1038/s41598-024-67407-w (PMC11252301; doi:10.1038/s41598-024-67407-w)
Supplement: Supplementary file 1 — Supplementary Tables. [file 41598_2024_67407_MOESM1_ESM.pdf]

Szota, K., van der Meer, A. S., Bourdeau, T., Chorpita, B. F., Chavanon, M.-L., & Christiansen, H. Pilot study of implementing Managing and Adapting Practice in a German psychotherapy master's program. *Scientific Reports.*

Supplemental material 1. Items and translations of all scales

Table S1. Perceived Characteristics of Intervention Scale (PCIS, Cook et al., 2015)

**Original**	**German translation**
1. [The treatment] is more effective than other therapies I have used.	1. [Die Behandlung] ist wirksamer als andere Therapien, die ich angewendet habe.
2. [The treatment] is more convenient than other therapies I have used.	2. [Die Behandlung] ist bequemer als andere Therapien, die ich angewendet habe.
3. Using [the treatment] fits well with the way I like to work.	3. Die Anwendung von [der Behandlung] passt gut zu meiner Art zu arbeiten.
4. [The treatment] is aligned with my clinical judgment.	4. [Die Behandlung] stimmt mit meinem klinischen Urteil überein.
5. [The treatment] is clear and understandable.	5. [Die Behandlung] ist klar und verständlich.
6. [The treatment] is easy to use.	6. [Die Behandlung] ist einfach zu handhaben.
7. [The treatment] can be tested out with patients without disrupting their overall therapy.	7. [Die Behandlung] kann mit Patient:innen ausprobiert werden, ohne ihre Behandlung im Gesamten zu unterbrechen.
8. It is easy to try out [the treatment] and see how it performs.	8. Es ist einfach, [die Behandlung] auszuprobieren und zu sehen, wie sie wirkt.
9. It is easy to tell whether patients are benefitting from [the treatment].	9. Es ist leicht zu erkennen, ob die Patient:innen von [der Behandlung] profitieren.
10. [The treatment] produces improvements in my patients that I can actually see.	10. [Die Behandlung] führt zu Verbesserungen bei meinen Patient:innen, die ich tatsächlich beobachten kann.
11. [The treatment] can be adapted to fit my treatment setting.	11. [Die Behandlung] kann an mein Behandlungssetting angepasst werden.
12. [The treatment] can be adapted to meet the needs of my patients.	12. [Die Behandlung] kann an die Bedürfnisse meiner Patient:innen angepasst werden.
13. Using [the treatment] improves the quality of work that I do.	13. Die Anwendung [der Behandlung] verbessert die Qualität meiner Arbeit.
14. Using [the treatment] makes it easier to do my job.	14. Die Anwendung [der Behandlung] erleichtert mir meine Arbeit.
15. The knowledge required to learn [the treatment] can be effectively taught.	15. Das Wissen, das zum Erlernen [des Verfahrens] erforderlich ist, kann effektiv vermittelt werden.
16. The skills required to implement [the treatment] can be effectively taught.	16. Die für die Durchführung [des Verfahrens] erforderlichen Fähigkeiten können wirksam vermittelt werden.
17. [The treatment] manual is helpful.	17. Das Handbuch [der Behandlung] ist hilfreich.
18. [The treatment] has helpful supportive materials for patients.	18. [Die Behandlung] hat hilfreiche Materialien zur Unterstützung der Patient:innen.

Table S2. Global assessments on confidence to use EBP

**Original (German)**	**English translation**
1. Wie beurteilen Sie Ihre Kompetenzen zu EBP?	1. How would you rate your competencies in EBP?
2. Wie beurteilen Sie Ihre Kenntnisse zu EBP?	2. How would you rate your knowledge of EBP?
3. Wie beurteilen Sie Ihre Fähigkeiten zum Einsatz von EBP?	3. How would you rate your ability to use EBP?
4. In welchem Ausmaß fühlen Sie sich für den Einsatz von EBP vorbereitet?	4. To what extent do you feel prepared to use EBP?
5. In welchem Ausmaß fühlen Sie sich für den Einsatz von EBP bereit?	5. To what extent do you feel ready to use EBP?
6. In welchem Ausmaß trauen Sie sich den Einsatz von EBP zu?	6. To what extent do you feel confident to use EBP?
